# Temporal Trends in Antibiotic Resistance of *Klebsiella pneumoniae* and Antibiotic Consumption: A Six-year Longitudinal Surveillance Study: Temporal Trends of Resistance and Consumption of Antibiotics to *K. pneumoniae*

**DOI:** 10.1007/s44197-026-00553-8

**Published:** 2026-04-15

**Authors:** Divya Bhat, Asha K Rajan, Neeraja Raju, Vandana Kalwaje Eshwara, Muralidhar Varma, Shashikiran Umakanth, Girish Thunga, Vishal Shanbhag

**Affiliations:** 1 Department of Critical Care Medicine, Kasturba Medical College, Manipal Academy of Higher Education, Manipal, Karnataka India; 2 Department of Pharmacy Practice Manipal College of Pharmaceutical Sciences, Manipal Academy of Higher Education, Manipal, Karnataka India; 3 Department of Microbiology, Kasturba Medical College, Manipal Academy of Higher Education, Manipal, Karnataka India; 4 Department of Infectious Diseases, Kasturba Medical College, Manipal Academy of Higher Education, Manipal, Karnataka India; 5 Department of General Medicine, Dr. TMA Pai Hospital, Udupi, Karnataka India

**Keywords:** *Klebsiella pneumoniae*, bacteraemia, antimicrobial resistance, antibiotic consumption, antimicrobial susceptibility

## Abstract

**Background:**

Antimicrobial resistance in *Klebsiella pneumoniae* represents a major challenge to healthcare systems. While antibiotic consumption is a key driver of resistance, long-term, setting-specific data linking antimicrobial use with resistance trends remain limited. This study aimed to evaluate temporal trends in antibiotic consumption and corresponding resistance patterns in *K. pneumoniae* over a six-year period.

**Methods:**

This retrospective, longitudinal surveillance study was conducted at a tertiary-care hospital from January 2019 to December 2024. Non-duplicate *K. pneumoniae* isolates recovered from various specimens were included. Antimicrobial susceptibility testing was performed using standardized laboratory methods and interpreted accordingly. Antibiotic consumptions data were extracted from electronic records, and quantified annually, with stratification by ICU and non-ICU settings. Temporal trends in resistance and consumption were analysed descriptively using various graphical representation.

**Results:**

Over the time, antibiotic consumption demonstrated a progressive shift toward broader spectrum and reserve agents, with a sustained increase in carbapenem use and persistently high fluoroquinolone utilization. Fluoroquinolone susceptibility remained uniformly low despite continued high use, whereas carbapenem susceptibility showed temporal fluctuations with an overall declining trend in later years. A transient improvement in susceptibility across multiple classes was observed in 2021, followed by renewed erosion thereafter. In contrast, aminoglycosides and tigecycline retained relatively stable activity, paralleling their restrained use.

**Conclusion:**

This six-year surveillance study highlights parallel temporal trends between antibiotic consumption patterns and evolving resistance in *K. pneumoniae*, with pronounced effects in critical care and bloodstream infections. Integrating consumption data with resistance surveillance is essential to inform setting-specific antimicrobial stewardship strategies and preserve the effectiveness of existing antibiotics in high-resistance healthcare environments.

**Supplementary Information:**

The online version contains supplementary material available at 10.1007/s44197-026-00553-8.

## Highlights

A six-year longitudinal surveillance study evaluated antibiotic consumption and resistance trends in *Klebsiella pneumoniae*.

Carbapenem use increased over time, with corresponding declines in susceptibility particularly in ICU bloodstream infections.

Fluoroquinolone utilization remained high despite persistently low *K. pneumoniae* susceptibility across settings.

Bloodstream and ICU isolates exhibited consistently higher resistance compared with non-invasive and ward isolates.

Restrained use of aminoglycosides and tigecycline was associated with relatively preserved antimicrobial activity.

**Supplementary Information:**

The online version contains supplementary material available at 10.1007/s44197-026-00553-8.

## Introduction

Antimicrobial resistance (AMR) has emerged as one of the most critical threats to global health, jeopardizing the effective treatment of infectious diseases and reversing decades of therapeutic progress [1]. The World Health Organization (WHO) has identified AMR as one of the top ten global health threats, given its profound impact on patient outcomes, healthcare costs, and health system sustainability [2–4]. Resistant bacterial infections are associated with prolonged illness, increased risk of complications, extended hospital stays, and substantially higher mortality rates [5]. In 2019 alone, bacterial AMR was associated with an estimated 4.95 million deaths worldwide, of which 1.27 million were directly attributable to resistant infections, underscoring the magnitude of this crisis [6]. The burden of AMR is disproportionately borne by low and middle-income countries (LMICs), which collectively account for approximately 85% of the global population. Nearly 40% of this population resides in the BRICS nations (Brazil, Russia, India, China and South Africa), regions that together contribute to more than four million AMR-associated deaths annually [7, 8]. In these settings, structural vulnerabilities, including overcrowded healthcare facilities, limited diagnostic capacity, inadequate infection prevention and control measures, widespread empirical antibiotic use, and weak regulatory oversight of antimicrobial prescribing, create an environment conductive to the emergence and dissemination of multi-drug resistant (MDR) pathogens [7, 9, 10]. Additional selective pressures arising from antibiotic use in agriculture and animal husbandry further compound this problem, facilitating the circulation of resistance determinants across human, animal and environmental interfaces [11]. India represents one of the largest contributors to the global AMR burden, with high rates of resistance reported among key gram-negative pathogens, including *K. pneumoniae*. Surveillance data from India have demonstrated increasing resistance to third-generation cephalosporins, fluoroquinolones and carbapenems, particularly in tertiary-care settings [12, 13]. Contributing factors include widespread empirical antibiotic use, limited antimicrobial stewardship implementation, and high burden of healthcare-associated infections. These challenges underscore the need for institution-specific surveillance studies to inform contextually relevant antimicrobial stewardship strategies in the Indian setting [14, 15].

Within hospital settings, gram-negative bacilli have become the predominant cause of healthcare-associated infections, particularly in intensive care units (ICUs), where patients are exposed to invasive devices, prolonged hospitalisation, and high-intensity antibiotic therapy. In such high-risk environments, deficiencies in antimicrobial stewardship (AMS) amplify selective pressure, accelerating the emergence of resistant organisms [16]. Among gram-negative pathogens, *Klebsiella pneumoniae* has gained particular prominence due to its remarkable capacity to acquire resistance mechanisms and cause severe infections, including pneumonia, bloodstream infections, urinary tract infections, intra-abdominal and hepatic abscesses [17–19]. Of greatest concern is the global rise of carbapenem-resistant *Klebsiella pneumoniae* (CRKP), which has been designated by the WHO as a critical priority pathogen [20]. CRKP poses a formidable clinical challenge owing to resistance to multiple antibiotic classes, including last-resort agents, and is associated with alarmingly high mortality rates, commonly ranging from 33% to 50% [18, 21–25]. Bloodstream infections caused by CRKP are especially devastating, frequently resulting in prolonged ICU stays, increased need for organ support, excess healthcare costs, and poor clinical outcomes [26]. In regions with high endemic resistance, clinicians are often compelled to rely on broad-spectrum or reserve antibiotics, further perpetuating the cycle of resistance.

Although antibiotic exposure is widely recognized as the single most important driver of AMR, the relationship between antimicrobial consumption and resistance evolution is complex, dynamic and highly context dependent. Resistance does not emerge uniformly across antibiotic classes, nor does it evolve in a linear fashion over time. Instead, it is shaped by local prescribing behaviours, infection control practices, patient case-mix, and healthcare setting [27]. Consequently, understanding temporal and setting-specific associations between antibiotic consumption and resistance patterns is essential for designing effective antimicrobial stewardship interventions. However, existing evidence is limited in several important aspects. Many surveillance studies report resistance trends without integrating contemporaneous antibiotic consumption data, thereby failing to capture the selective pressures driving these trends. Others are restricted to short observation periods, focus on single antibiotic classes, or do not differentiate between ICU and non-ICU settings, despite clear differences in antimicrobial exposure and resistance epidemiology [28–31].

Therefore, this retrospective observational study was undertaken to examine long-term trends in antibiotic consumption across ICUs and wards, to characterize temporal changes in antimicrobial susceptibility among *K. pneumoniae* isolates from key clinical sources, and to explore the ecological relationships between antibiotic use and resistance patterns over a six-year period. By contextualizing resistance trends within contemporaneous prescribing practices, this study aims to provide evidence that can inform targeted antimicrobial stewardship strategies, support rational empirical therapy, and ultimately contribute to mitigating the escalating burden of AMR in high-risk hospital settings.

## Methods

### Ethical Statement

This study was reviewed and approved by the Institutional Ethics Committee of the study centre [IEC approval number:5/2024]. Given the retrospective and observational nature of the study, the requirement for informed consent was waived. All patient identifiers were removed prior to data extraction, and analyses were performed on fully anonymised datasets in accordance with institutional policies and national ethical guidelines governing retrospective research involving human participants.

### Study Design, Setting and Population

This was a retrospective, observational surveillance study conducted at Kasturba Hospital, Manipal, a tertiary-care academic centre with approximately 2,032 in-patient beds. The hospital caters to an average of 3000 outpatients daily, with approximately 200–250 patients admitted each day. Critical care department manages 32 intensive care unit beds and 14 high dependency unit beds. The hospital comprises multiple specialized ICU, including medical, surgical and critical care units, catering to a wide range of patient populations. Infectious disease consultation services are available within the institution, supported by a multidisciplinary team including ID physicians, microbiologists and clinical pharmacists. The hospital has an established antimicrobial stewardship program (ASP), which operates through prospective audit and feedback, formulary restriction of selected antibiotics, and implementation of institutional antibiotic guidelines. The ASP is integrated with the hospital infection control committee and is periodically updated based on local antimicrobial resistance surveillance data. The antimicrobial stewardship program has been operational since 2015, providing structured oversight of antimicrobial prescribing practices within the institution.

The study encompassed a six-year period from January 2019 and December 2024, allowing evaluation of long-term temporal trends in antimicrobial resistance and antibiotic consumption across pre-pandemic, pandemic and post-pandemic phases. Clinical isolates were defined as non-duplicate *K. pneumoniae* isolates obtained from routine diagnostic specimens collected for clinical purposes. All such isolates recovered from hospitalized patients during the study period were eligible for inclusion. Isolates were obtained from routine diagnostic specimens, including blood, respiratory samples (such as sputum and endotracheal aspirates), urine and exudates (including wound swabs and other body fluid specimens). No additional specimen categories were excluded: wound and fluid samples were included under the exudate category for analytical consistency. To minimise bias from repeated sampling, only non-duplicate isolates were included, defined as the first isolate per patient per admission episode from a given specimen type. Both ICU and non-ICU ward settings were included to enable setting-specific analyses.

### Microbiological Processing and Antimicrobial Susceptibility Testing

All clinical specimens were processed in the hospital’s accredited microbiology laboratory following standard operating procedures. Blood cultures were performed by Bact/ALERT^®^ VIRTUO^®^ automated system (bioMérieux). Other clinical specimens were processed according to standard laboratory practices. Definitive species-level identification was confirmed using Matrix-Assisted Laser Desorption/Ionization Time-of-Flight (MALDI-TOF) mass spectrometry (Vitek-MS), ensuring consistent and accurate identification across the study period. All microbiological procedures were performed in accordance with standard laboratory operating protocols aligned with Clinical and Laboratory Standards Institute (CLSI) guidelines.

Antibiotic susceptibility testing (AST) was performed using the VITEK 2 Compact system (bioMérieux) with appropriate GN-AST cards, and interpreted using the CLSI guidelines applicable for the corresponding year of testing [32]. Although CLSI breakpoints may undergo periodic updates, the laboratory adhered consistently to year-specific CLSI standards throughout the study period, with no major institutional changes in testing methodology. The antimicrobial panel included: aminoglycosides (amikacin, gentamicin), β-lactam/β-lactamase inhibitor combinations (amoxicillin-clavulanate, piperacillin-tazobactam, cefoperazone-sulbactam), cephalosporins (cefuroxime, cefotaxime, ceftriaxone, cefepime), carbapenems (imipenem, meropenem), fluoroquinolones (ciprofloxacin), aztreonam, trimethoprim-sulfamethoxazole and fosfomycin. For temporal trend analysis and graphical representation, selected antibiotics were used as representatives of their respective classes (e.g. Ceftriaxone for third-generation cephalosporins and cefepime for fourth-generation cephalosporins, due to consistent data availability across the study period), based on completeness and consistency of data across the study period. Different graphical formats were used to best represent the data, with heatmaps employed to visualize multi-year susceptibility patterns across antibiotics and bar/line graphs used to highlight temporal trends in specific clinical settings. Antibiotics with incomplete longitudinal data (e.g. cefuroxime and cefotaxime) were not included in the final trend analyses.

### Resistance Surveillance Data Compilation

Isolate-level susceptibility data, including specimen source, care location (ICU or ward), and percentage susceptibility for each antimicrobial agent, were extracted from the laboratory information system. These data were compiled annually by the hospital infection control committee (HICC) as part of routine institutional antimicrobial resistance surveillance. For bloodstream isolates, complete year-wise susceptibility data were available across the entire six-year study period (2019–2024). For isolates from urine, respiratory samples, and exudates, consistent temporal susceptibility data were available from 2021 to 2024. All datasets were anonymized prior to analysis and formed for descriptive resistance trend evaluation.

### Antibiotic Consumption Data

Antibiotic consumption data for the same study period (January 2019 to December 2024) were retrieved from the hospital’s electronic medication and dispensing records through the Information Technology department. The raw dataset included inpatient identification number, antibiotic name, quantity dispensed, and location of administration (specific ward or ICU). ‘Quantity dispensed’ refers to the number of antibiotic units issued by the hospital pharmacy (e.g., vials, tablets or doses). For the purpose of this study, antibiotic consumption was quantified using the total number of prescriptions (dispensing events) per year to ensure consistency across different antibiotic formulations. Annual antibiotic consumption was quantified as the total number of units or prescriptions administered each year. Because denominator variables such as patient-days, admission volume, and bed occupancy were not consistently available across the entire six-year period, standardized metrics such as defined daily doses (DDD) or days of therapy (DOT) per 1000 patient-days could not be calculated retrospectively. Therefore, annual prescription counts were used to enable consistent temporal comparison of antibiotic utilization patterns within the same institution over time. Consumption data were aggregated at the institutional level and stratified by ICU and non-ICU settings to capture differences in prescribing intensity across care locations. Antibiotics were categorized into mechanistic classes to facilitate interpretation of class-level prescribing trends in relation to antimicrobial resistance patterns. Although WHO ATC/DDD classification provides a standardized framework for drug utilization studies, mechanistic grouping was used in this study to support ecological comparison of antibiotic classes within the institution. This included:

i. β-Lactam antibiotics, further subclassified into penicillin, cephalosporins, carbapenems, and monobactams.
ii. Protein Synthesis Inhibitors, including tetracyclines and aminoglycosides.
iii. Nucleic Acid Synthesis Inhibitors, primarily fluoroquinolones.
iv. Non- β-Lactam Cell Wall Synthesis Inhibitors)
v. Antimetabolites and reserve agents, including trimethoprim-sulfamethoxazole, fosfomycin, polymyxin B, and colistin, which were also analyzed individually due to their clinical relevance in multidrug-resistant infections. Fosfomycin utilization included intravenous and oral formulations as recorded in pharmacy dispensing data.

### Outcomes and Analytical Approach

The study evaluated two primary outcomes. The first outcome was antimicrobial resistance, expressed as the annual percentage of *K. pneumoniae* isolates susceptible to each antibiotic. Resistance trends were assessed for the overall hospital population and stratified by clinical setting (ICU versus non-ICU) and specimen source (bloodstream, respiratory tract, urinary tract, and exudate). The second outcome was antibiotic consumption, measured as annual utilization counts for individual antibiotics and mechanistic classes, with stratification by ICU and ward locations. The analytical approach was primarily descriptive and exploratory, consistent with the ecological design of the study. Temporal trends in antimicrobial consumption and susceptibility were visualized using line graphs and heatmaps. Dual-axis temporal plots were employed to visually explore the relationship between antibiotic consumption and corresponding susceptibility trends for major antibiotic classes, including carbapenems, fluoroquinolones, and third-generation cephalosporins. Given the ecological design and aggregated nature of the data, the analysis was descriptive and exploratory, and no formal regression modelling or statistical testing was performed. This approach enabled assessment of concordant and discordant patterns between prescribing intensity and resistance evolution across different clinical settings over time. Data analysis was primarily descriptive and was performed using Microsoft Excel. Temporal trends were visualized using line graphs, heatmaps and dual-axis plots.

## Results

### Trends in Antibiotic Consumption over Six years (2019–2024)

**Fig. 1:**
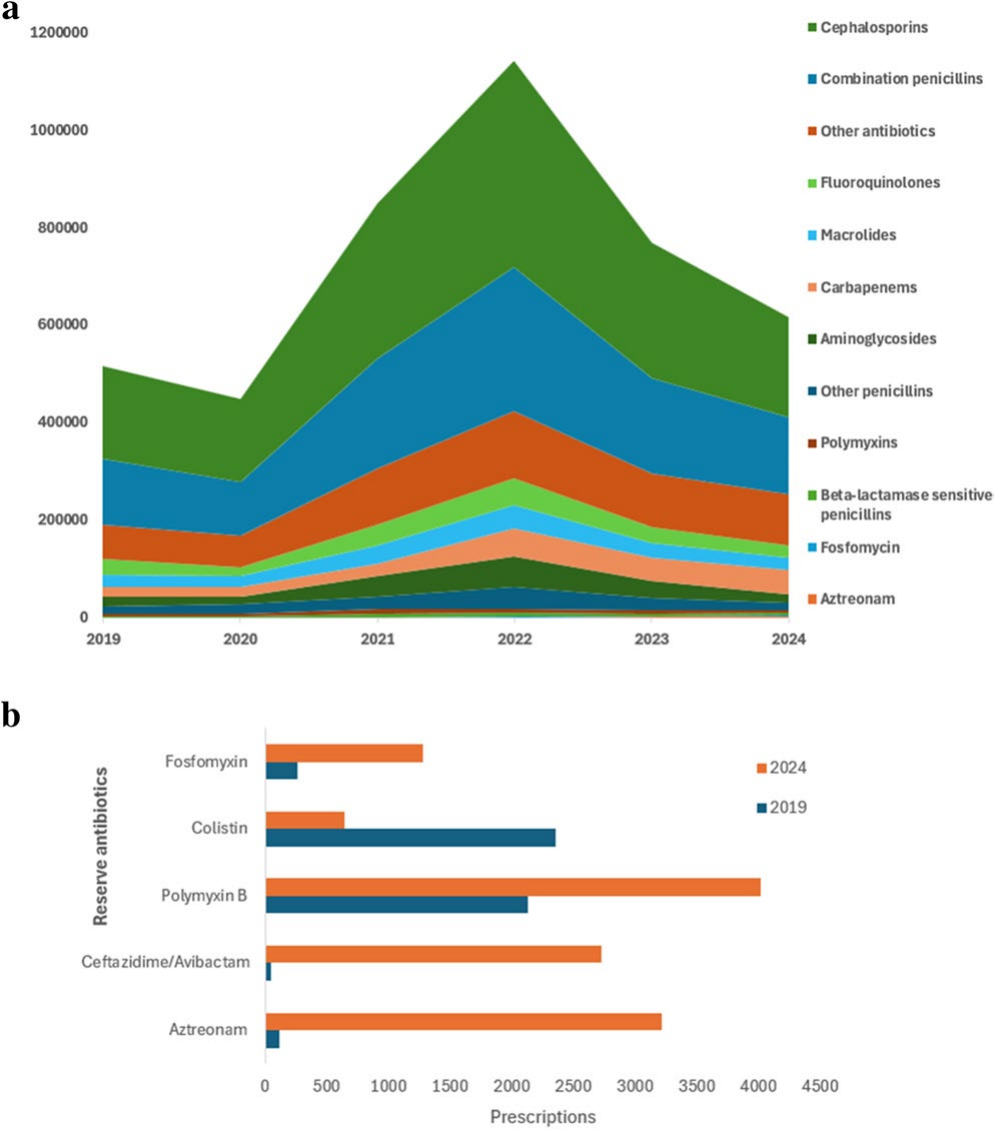
**a** Number of antibiotic prescriptions stratified by antibiotic class for each year **b** Changes in utilization of reserve antibiotics between 2019 and 2024

Temporal patterns in antibiotic utilization across major antimicrobial classes are summarized in Fig. 1a. Over the six-year observation period, a progressive shift toward broader-spectrum and reserve antibiotics was evident at the institutional level. Carbapenem consumption demonstrated an overall increasing trend over time, with a peak observed around 2022 followed by a relative decline in subsequent years. This escalation was particularly marked in ICUs, reflecting increased reliance on reserve β-lactam agents in the management of severe and drug-resistant infections, although a parallel upward trend was also observed in non-ICU settings. Fluoroquinolone use remained consistently sustained throughout the study period. Although absolute consumption was lower than β-lactam antibiotics, its continued use across all years despite low susceptibility highlights persistent prescribing of this class. Despite contemporaneous changes in antimicrobial susceptibility, fluoroquinolones continued to account for a substantial proportion of overall antibiotic prescriptions, indicating sustained empirical use across multiple clinical syndromes. In contrast, third-generation cephalosporin utilization (represented by ceftriaxone) was highest during the earlier years of the study and subsequently demonstrated relative stabilization or a modest decline in later years. This temporal pattern coincided with increasing carbapenem use, suggesting a gradual shift away from traditional first-line β-lactams agents toward broader-spectrum alternatives. Utilization of β-lactam/β-lactamase inhibitor combinations exhibited variable trends over time, characterised by intermittent increases and decreases. These fluctuations likely reflect evolving empirical prescribing strategies aimed at balancing antimicrobial coverage with stewardship considerations. Aminoglycosides use remained comparatively low and stable throughout the study period, consistent with their predominantly adjunctive or targeted role in clinical practice.

Analysis of reserve and newer antimicrobial agents revealed marked changes between 2019 and 2024 (Fig. 1b). Within the polymyxin class, a clear decline in colistin use was observed, accompanied by a substantial increase in polymyxin B utilization. Concurrently, fosfomycin use increased progressively over time. Notably utilization of newer β-lactam/β-lactamase inhibitor combinations and monobactams, including ceftazidime/avibactam and aztreonam, also increased, reflecting the adoption of alternative therapeutic options in response to evolving resistance patterns. Collectively, these data demonstrate a progressive broadening of antimicrobial exposure over time, characterised by increasing reliance on carbapenems, persistently high fluoroquinolone use and dynamic reallocation of reserve agents.

### Source-stratified Susceptibility Profiles of *K. pneumoniae* in ICU and Non-ICU Settings

Year-wise antimicrobial susceptibility profiles of ICU-derived *K. pneumoniae* isolates stratified by clinical source are presented in Supplementary figures S1a-d. There was a total of 1,367 *K. pneumoniae* isolates from blood across all years. Bloodstream isolates consistently exhibited lower susceptibility to multiple antibiotic classes compared with isolates obtained from respiratory, urinary and exudate specimens. Reductions in susceptibility were most pronounced for cephalosporins and fluroquinolones, whereas carbapenems and aminoglycosides generally retained comparatively higher activity, albeit with temporal variability. Supplementary figures S2 presents a heatmap of antimicrobial susceptibility among non-ICU *K. pneumoniae* isolates across clinical sources. In comparison with ICU isolates, non-ICU isolates generally exhibited higher susceptibility across most antibiotic classes. However, reduced susceptibility to fluoroquinolones and third-generation cephalosporins was observed across multiple specimen types, indicating dissemination of resistance beyond critical care environments.

### Temporal Trends in *K. pneumoniae* Susceptibility in Bloodstream Infections by Clinical Setting

**Fig. 2:**
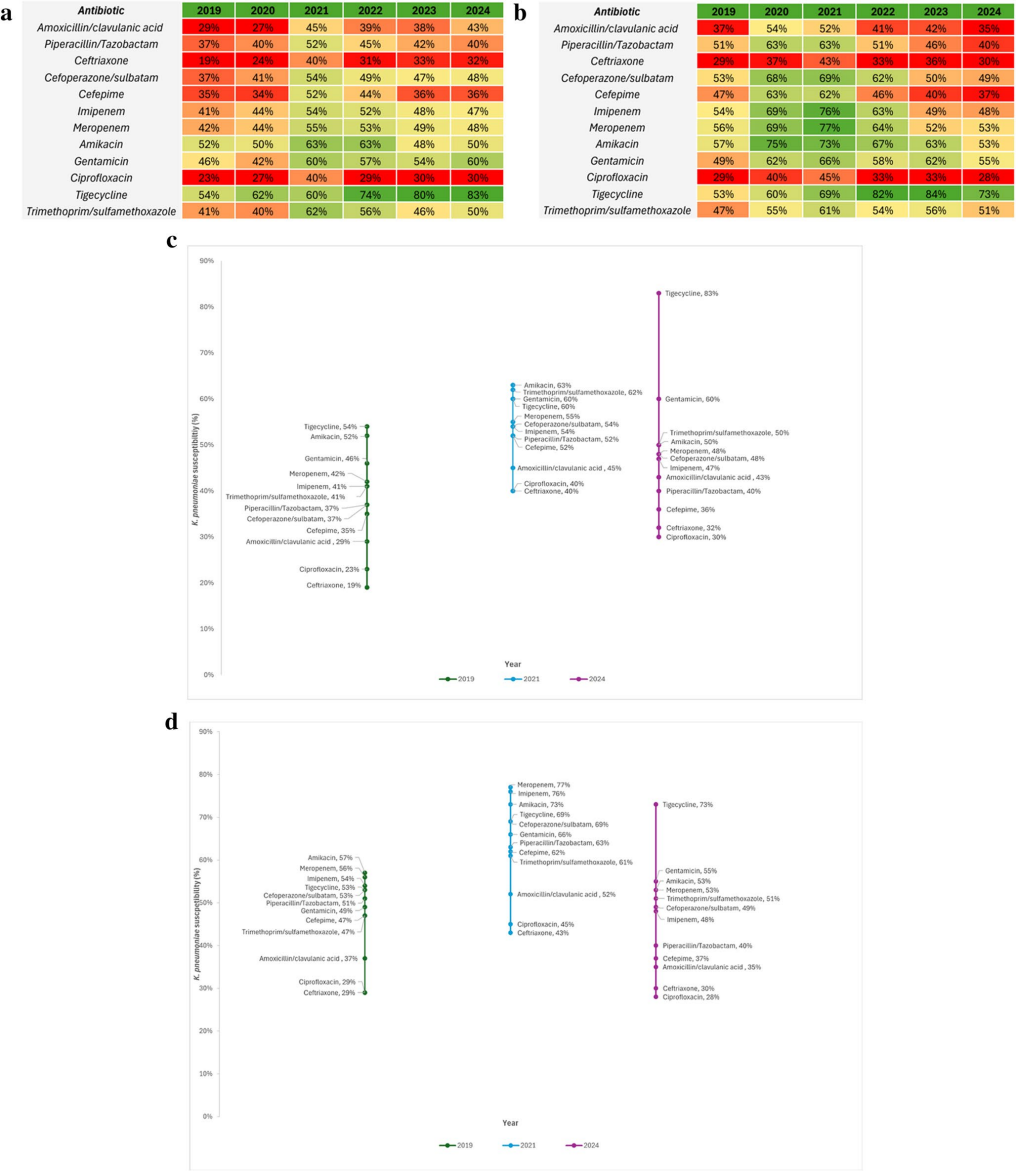
**a** Temporal heatmap of antimicrobial susceptibility of *Klebsiella pneumoniae* bloodstream isolates in Intensive care units **b** Temporal heatmap of antimicrobial susceptibility of *Klebsiella pneumoniae* bloodstream isolates in non-ICU **c** Antimicrobial susceptibility of *Klebsiella pneumoniae* bloodstream isolates in ICU settings across selected study years (2019, 2021 & 2024) **d** Antimicrobial susceptibility of *Klebsiella pneumoniae* bloodstream isolates in ward settings across selected study years (2019, 2021 & 2024). Each cell displays the percentage susceptibility of *Klebsiella pneumoniae* isolates to the corresponding antibiotic for the given year. The color gradient provides a visual representation of susceptibility levels, with red shades indicating lower susceptibility and green shades indicating higher susceptibility

Given that bloodstream isolates demonstrated the poorest susceptibility across sources, a focused analysis of *K. pneumoniae* bloodstream infections was undertaken to characterise temporal trends by clinical setting. Year-wise susceptibility heatmaps for ICU and non-ICU bloodstream isolates are shown in Fig. 2a and b, with selected-year comparisons (2019, 2021, and 2024), chosen to represent baseline (pre-pandemic), midpoint, and the most recent study year; presented in Fig. 2c and d. Among ICU bloodstream isolates, susceptibility to multiple β-lactam agents, including third-generation cephalosporins, represented by ceftriaxone, and to fluoroquinolones, represented by ciprofloxacin, declined over time. In contrast, susceptibility to carbapenems and selected reserve agents remained comparatively preserved but exhibited substantial year-to-year variability. Notably, a distinct peak in susceptibility across several antibiotic classes was observed around 2021, followed by a decline in subsequent years. This temporal progression is clearly illustrated in the longitudinal comparison (Fig. 2c and d), which demonstrates a marked reduction in susceptibility to most agents between 2019 and 2024.

In non-ICU wards, bloodstream isolate susceptibility patterns were generally more stable overtime. Nevertheless, gradual declines in susceptibility to fluoroquinolones and cephalosporins were evident. Similar to ICU isolate, peak susceptibility for several agents was observed around 2021, followed by stabilization or decline thereafter. Across all years, susceptibility to carbapenem and aminoglycoside, represented by amikacin, remained consistently higher in ward isolates compared with ICU isolates. Across both ICU and non-ICU settings, aminoglycosides and tigecycline retained higher in vitro activity relative to other antibiotic classes. However, these agents are not preferred first-line therapies for bloodstream infections due to pharmacokinetic limitations and potential toxicity and are typically used in selected clinical scenarios or as part of combination regimens.

### Association Between Antibiotic Consumption and *K. pneumoniae* Susceptibility in Bloodstream Infections

**Fig. 3:**
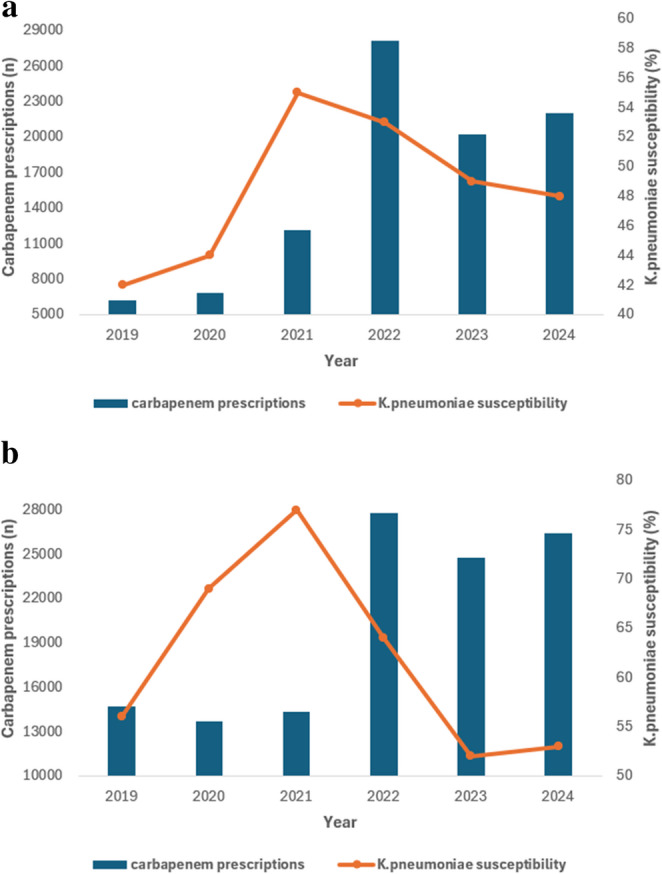
**a** Temporal trends in carbapenem utilization and *K. pneumoniae* susceptibility among ICU bloodstream infections **b** Temporal trends in carbapenem utilization and *K. pneumoniae* susceptibility among non-ICU bloodstream infections

**Fig. 4:**
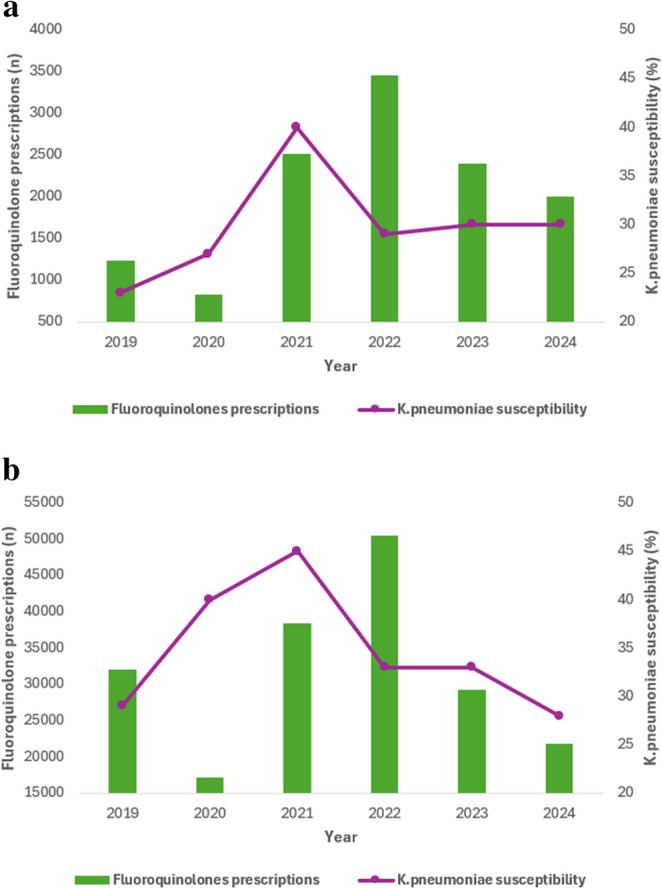
**a** Temporal trends in fluoroquinolone utilization and K. pneumoniae susceptibility among ICU bloodstream infections **b** Temporal trends in fluoroquinolone utilization and *K. pneumoniae* susceptibility among non-ICU bloodstream infections

**Fig. 5:**
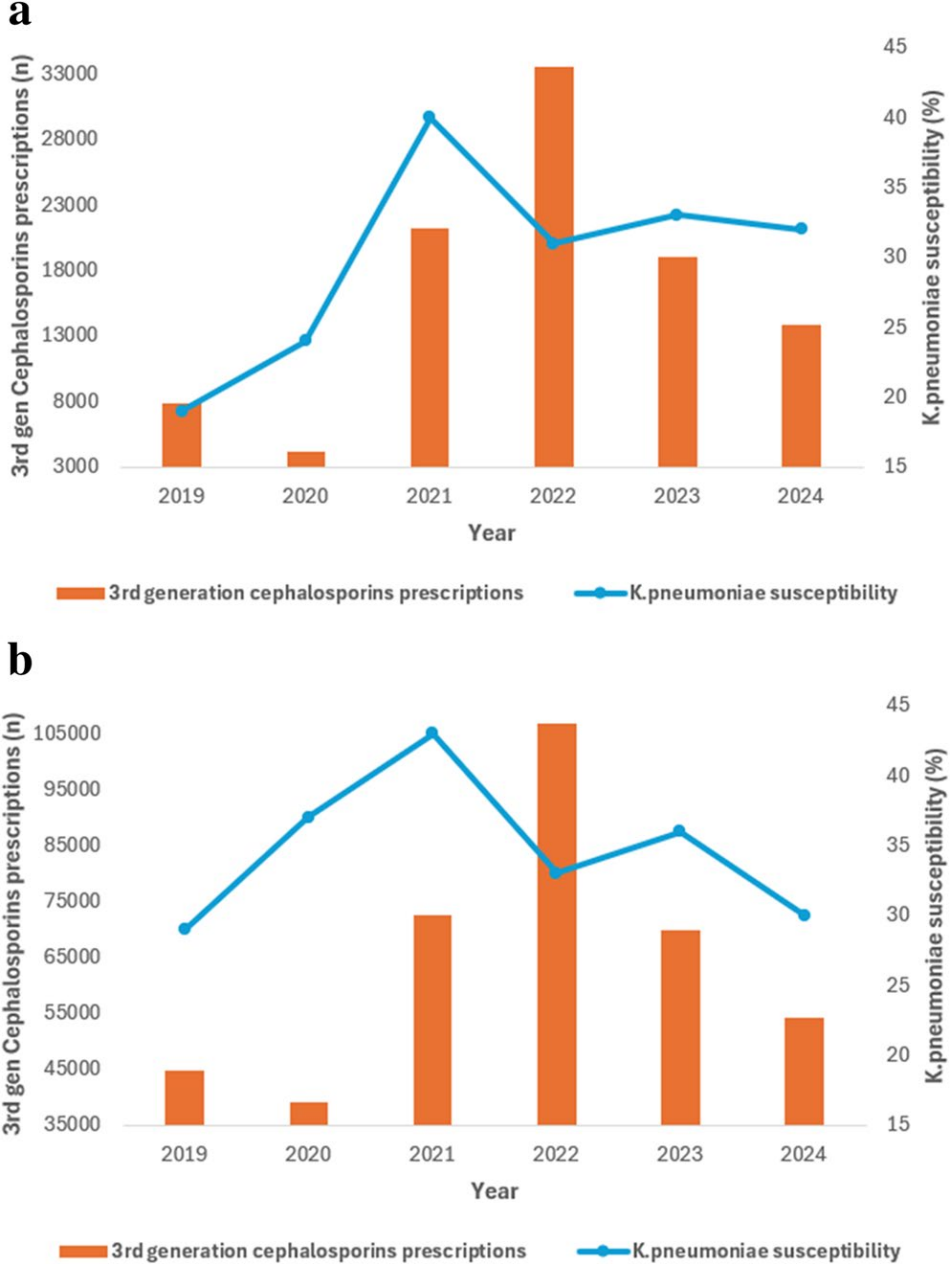
**a** Temporal trends in 3rd generation cephalosporins utilization and *K. pneumoniae* susceptibility among ICU bloodstream infections **b** Temporal trends in 3rd generation cephalosporins utilization and *K. pneumoniae* susceptibility among non-ICU bloodstream infections. These dual-axis graphs illustrate temporal trends in antibiotic utilization and *Klebsiella pneumoniae* susceptibility over the study period. The graphical comparisons are descriptive and intended to visually explore temporal patterns; no formal statistical association between antibiotic consumption and susceptibility was tested

Dual-axis temporal analyses exploring the relationship between antibiotic consumption and susceptibility among bloodstream isolates are presented in Figs. 3, 4 and 5. Increasing carbapenem utilization over time was observed in both ICU and non-ICU settings (Fig. 3a and b). This increase coincided with fluctuating susceptibility patterns, with susceptibility peaking around 2021 and subsequently declining, particularly among ICU isolates. Although carbapenem susceptibility remained higher than that of several other antibiotic classes, a divergence between rising utilization and attenuated susceptibility became evident by 2024 in both care settings. While susceptibility remained relatively higher compared with other antibiotic classes, the divergence between rising utilization and attenuated susceptibility visually suggests evolving selective pressure in bloodstream isolates. In non-ICU wards, carbapenem consumption also increased progressively. Corresponding susceptibility trends were relatively stable in the earlier years but demonstrated a gradual decline in later years, broadly mirroring the rising consumption curve. The temporal relationship between fluoroquinolone utilization and *K. pneumoniae* susceptibility in bloodstream infections is shown in Fig. 4a and b. Fluoroquinolone use remained consistently high throughout the study period, while susceptibility to ciprofloxacin remained persistently low, with minimal recovery in both ICU and non-ICU bloodstream isolates. Temporal trends for third-generation cephalosporins are presented in Fig. 5a and b. Utilization of these agents remained substantial, particularly in the earlier years of the study. Susceptibility to ceftriaxone and related cephalosporins peaked around 2021 and declined thereafter, with the reduction more pronounced among ICU bloodstream isolates. These patterns are consistent with ongoing selection pressure on extended-spectrum β-lactamase-producing *K. pneumoniae*.

### Temporal trends in Other Clinical Specimen Sources

Temporal analyses of *K. pneumoniae* isolates from respiratory, urinary tract, and exudate specimens broadly similar relationships between antibiotic consumption and susceptibility, although the magnitude and rate of resistance varied by source (Supplementary figures S3-10). Among respiratory isolates increasing carbapenem utilization was associated with declining carbapenem susceptibility over time. Sustained high use of fluoroquinolones and piperacillin-tazobactam, particularly in non-ICU settings, was accompanied by progressive reductions in susceptibility to these agents. These trends were consistently more pronounced in ICU-derived respiratory isolates than in those from wards. In urinary tract infections, persistently high fluoroquinolone utilization corresponds with low susceptibility, while continued use of third-generation cephalosporins was associated with declining effectiveness over time. Among exudate-derived isolates, increased carbapenem use was again associated with reduced susceptibility. In contrast, susceptibility to aminoglycosides and piperacillin-tazobactam remained relatively stable, consistent with their more restricted or targeted use.

## Discussion

This longitudinal surveillance study delineates a complex and evolving relationship between antimicrobial consumption and resistance in *K. pneumoniae*, characterized by pronounced class-specific, setting-dependent, and time-sensitive trends. Rather than a uniform escalation of resistance across all antibiotic classes, the findings reveal a heterogenous resistance landscape shaped by differential prescribing pressures, clinical context, and stewardship practices. Collectively, the data depicts a healthcare system operating under sustained selective pressure, in which changes in antibiotic utilization are reflected in resistance patterns, albeit with varying degrees of responsiveness across drug classes. Importantly, the observed trends indicate not merely a quantitative change in antibiotic use but a qualitative reorganisation of institutional prescribing strategies in response to evolving resistance epidemiology. The most notable shift was the marked increase in the utilization of newer, targeted agents, particularly ceftazidime-avibactam and aztreonam, by 2024 compared with 2019, accompanied by a relative reduction in the use of extended-spectrum carbapenems. The gradual de-escalation of meropenem use and the early withdrawal of imipenem, contrast with reports from several centres that documented increased carbapenem consumption during the COVID-19 pandemic [33, 34]. This divergence highlights the heterogeneity of AMS responses across institutions and underscores the importance of local epidemiology in shaping prescribing behaviour [34, 35].

The increase in ceftazidime-avibactam and aztreonam use in our setting appears to reflect a rational, adaptive response to the regional predominance of Metallo-β lactamase-producing carbapenem-resistant Enterobacterales. The timely adoption of guideline-endorsed combination strategies, as recommended by international and national bodies, suggests a deliberate shift away from older, more toxic regimens reliant on polymyxins and aminoglycosides [36–39]. In parallel, the stable and controlled use of cornerstone agents such as piperacillin-tazobactam and amikacin further supports the presence of an intentional, stewardship-driven approach rather than reactive escalation. Taken together, these consumption patterns portray a health system that progressively transitioned toward precision-based antimicrobial use, guided by evolving evidence and resistance profiles. Interpretation of temporal resistance trends must also account for the profound impact of the COVID-19 pandemic. A striking observation in this study was the uniform improvement in antimicrobial susceptibility across multiple antibiotic classes in 2021. This transient “resistance reprieve” temporally coincided with the peak of pandemic-related infection prevention and control interventions, including enhanced hand hygiene, universal masking, reduced inter-ward patient movement, and the establishment of dedicated COVID-19 care units. These measures likely curtailed horizontal transmission of nosocomial *K. pneumoniae* clones. Additionally, heightened diagnostic vigilance and cautious antibiotic prescribing in non-COVID patients may have contributed to reduced selective pressure. Similar temporary declines in MDR infections have been reported elsewhere during periods of intensified infection control [40, 41]. 

The subsequent decline in susceptibility observed from 2022 onwards suggests that these gains were not sustained once pandemic-related measures were relaxed and routine hospital dynamics resumed. The sustained high utilization of fluoroquinolones, despite persistently low susceptibility, highlights a clear disconnect between prescribing practices and microbiological effectiveness. This pattern represents chronic, low-intensity selective pressure sufficient to maintain resistant *K. pneumoniae* populations without triggering a meaningful therapeutic shift. This pattern deviates from the classical “use-resistance-abandonment” cycle and may reflect a mismatch between antimicrobial utilization and organ-specific susceptibility trends within the local context. However, it is important to acknowledge that fluoroquinolones gave broader clinical indications, including use for atypical respiratory pathogens and selected gram-negative infections, which may contribute to their sustained utilization. Therefore, the observed discordance should be interpreted within the context of overall prescribing practices rather than being solely attributed to inappropriate use for *K. pneumoniae* infections. Fluoroquinolones in our institution are prescribed according to standard dosing regimens as per institutional antibiotic guidelines, with adjustments based on patient-specific factors such as renal function. However, detailed dosing analysis was beyond the scope of this study, which utilized aggregated consumption data. Persistently low ciprofloxacin susceptibility observed in this study aligns with resistance rates reported in comparable bloodstream infection cohorts, reinforcing the generalisability of this concern [42]. Progressive increases in carbapenem consumption, particularly within ICUs, were accompanied by fluctuating but overall declining susceptibility. These patterns are unlikely to represent random variation and instead suggest the early epidemiological phase of carbapenemase dissemination within a high-risk population. Comparable studies from tertiary-care settings have reported similarly high burdens of MDR and carbapenemase production among *K. pneumoniae* BSI, underscoring the endemic pressure faced by such institutions [42].

Third-generation cephalosporins displayed another classic resistance pattern, wherein sustained utilization was accompanied by progressive reductions in susceptibility, consistent with ongoing selection of extended-spectrum β-lactamase-producing strains. In contrast, aminoglycosides and tigecycline retained relatively preserved activity throughout the study period. According to the “antibiotic lifecycle” concept, the clinical longevity of an antimicrobial class can be extended through deliberate avoidance in empirical therapy and reserved deployment for well-defined indications. Despite operating in a high-resistance environment, susceptibility to amikacin and gentamicin in our setting remained relatively stable over time. Finally, setting and source-specific analyses provided additional insights into resistance evolution. ICU isolates consistently exhibited higher resistance across most Watch and Reserve antibiotic categories compared with ward isolates, underscoring the amplifying effect of critical care environments on resistance emergence. Similarly, bloodstream isolates demonstrated poorer susceptibility than isolates from respiratory, urinary or exudate sources, reinforcing the association between invasive infections, cumulative antimicrobial exposure, and resistance selection. Together, these findings outline a dynamic temporal resistance landscape in which escalating and sustained broad-spectrum antibiotic use drives differential resistance trajectories across drug classes, clinical settings and infection sources. Nevertheless, institution-specific surveillance studies such as this remain essential for guiding local antimicrobial stewardship interventions, as resistance patterns and antibiotic utilization practices often vary substantially between hospitals and geographic regions.

### Implications for clinical practice and antimicrobial stewardship

The findings of this study highlight the need for improved alignment between antimicrobial prescribing practices and local susceptibility patterns. In particular, the sustained use of fluoroquinolones despite low susceptibility underscores the importance of regularly updating empirical therapy guidelines based on institution-specific antibiograms. Similarly, emerging reductions in carbapenem susceptibility, especially in critical care settings, emphasize the need for carbapenem-sparing strategies where appropriate and early de-escalation based on microbiological data.

These observations support strengthening antimicrobial stewardship interventions, including prospective audit and feedback, periodic revision of institutional antibiotic policies, and enhanced integration of microbiological data into clinical decision-making. Future efforts should focus on incorporating standardized antibiotic utilization metrics and expanding surveillance frameworks to include multicentre data and patient-level analyses to better inform stewardship strategies.

### Strengths and Limitations

This study has several important strengths. First, it provides a longitudinal, six-year surveillance analysis integrating antimicrobial consumption with resistance patterns of *K. pneumoniae*, allowing assessment of temporal dynamics rather than static resistance snapshots. The analysis is granular and context-specific, with stratification by clinical setting (ICU versus non-ICU) and by infection source thereby capturing heterogeneity that is often masked in aggregate antibiogram-based reports. The use of routinely generated microbiological and prescribing data enhances the real-world relevance of the findings and strengthens their applicability to antimicrobial stewardship practice. Importantly, the concurrent evaluation of antibiotic utilization and susceptibility trends offers actionable insights that extend beyond descriptive resistance reporting.

The study also has few limitations which needs consideration. Most notably, the absence of molecular characterization of resistance mechanisms precludes definitive attribution of observed phenotypic trends to specific carbapenemase or extended-spectrum β-lactamases. As a result, resistance evolution could not be correlated with clonal dissemination or horizontal gene transfer events. Additionally, the single-centre design may limit the external validity and generalizability to the findings to other healthcare institutions with differing patient populations, antimicrobial stewardship practices, or resistance epidemiology. Although tertiary-care hospitals in many LMIC settings face similar antimicrobial resistance challenges, the observed trends should be interpreted within the context of the local institutional environment. In addition, periodic updates in CLSI breakpoint criteria over time may influence susceptibility categorization; however, year-specific standards were applied consistently, and no major methodological changes occurred during the study period. The ecological nature of the analysis also precludes causal inference between antibiotic consumption and resistance, as patient-level exposure-outcome relationships were not assessed. Furthermore, this study focused on a single organism, *K. pneumoniae*, and therefore does not capture resistance patterns across other clinically relevant pathogens. In addition, the use of aggregated, dispensing-based antibiotic consumption data precluded assessment of indication-specific prescribing, patient-level dosing regimens and the use of combination (dual or triple) antibiotic therapy. Consequently, the study could not evaluate the appropriateness or optimization of antibiotic use in relation to resistance development. Antibiotic consumption was quantified using prescription counts rather than standardized metrics such as DDD or DOT per 10,000 patient-days due to the absence of consistent denominator data across the study period. Future studies incorporating standardized consumption metrics would further strengthen comparisons across time and institutions. In addition, the study relied on descriptive ecological analyses without formal regression modelling or statistical testing to quantify the association between antibiotic consumption and resistance trends. Therefore, the findings should be interpreted as hypothesis-generating rather than evidence of causal relationships.

## Conclusion

This longitudinal surveillance study demonstrates that antimicrobial resistance in *K. pneumoniae* closely parallels patterns of antibiotic consumption, with distinct temporal and setting-specific effects. Sustained use of broad-spectrum agents, particularly fluoroquinolones was associated with persistently low susceptibility across clinical settings, whereas carbapenem susceptibility showed variable temporal trends, with more pronounced changes observed in bloodstream infections, especially outside critical care settings. In contrast restrained and targeted use of selected antibiotics was associated with relative preservation of activity. These findings highlight the critical importance of integrating antibiotic consumption data with resistance surveillance to guide empirical therapy and antimicrobial stewardship. Continuous, context-specific monitoring and timely policy adaptation are essential to preserve the effectiveness of existing antibiotics in high-resistance healthcare settings.

The figure provides an overview of antibiotic prescribing patterns across multiple antimicrobial classes; selected key classes are emphasized in the text based on their clinical relevance and resistance trends.

## Supplementary Information

Below is the link to the electronic supplementary material.

Supplementary Material 1 (DOCX 1.07 MB)

## Data Availability

All relevant data are within the manuscript and its supporting information files. The raw data sets of patients are available from the corresponding author upon request. Any additional data related to ethical details of the study are available from Kasturba Medical College and Kasturba Hospital Institutional Ethics Committee, email: [iec.kmc@manipal.edu](mailto: iec.kmc@manipal.edu).

## Declarations

### Transparency declarations

None to declare
