# Supplementary material for: Temporal Trends in Antibiotic Resistance of Klebsiella pneumoniae and Antibiotic Consumption: A Six-year Longitudinal Surveillance Study: Temporal Trends of Resistance and Consumption of Antibiotics to K. pneumoniae
Source: J Epidemiol Glob Health. 2026 Apr 15;16(1):61. doi: 10.1007/s44197-026-00553-8 (PMC13201805; doi:10.1007/s44197-026-00553-8)
Supplement: Supplementary file 1 — Supplementary Material 1 (DOCX 1.07 MB) [file 44197_2026_553_MOESM1_ESM.docx]

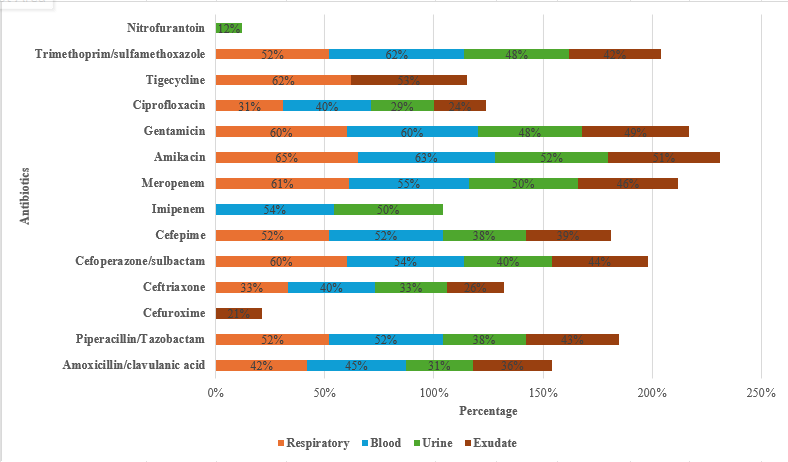


**Supplementary figure S1a: Year-wise antimicrobial susceptibility of *K. pneumoniae* isolated from various clinical sources in ICUs (2021)**


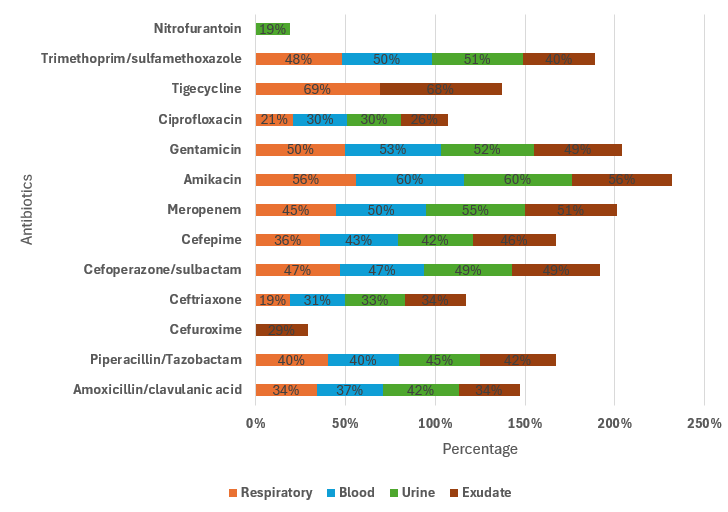


**Supplementary figure S1b: Year-wise antimicrobial susceptibility of *K. pneumoniae* isolated from various clinical sources in ICUs (2022)**


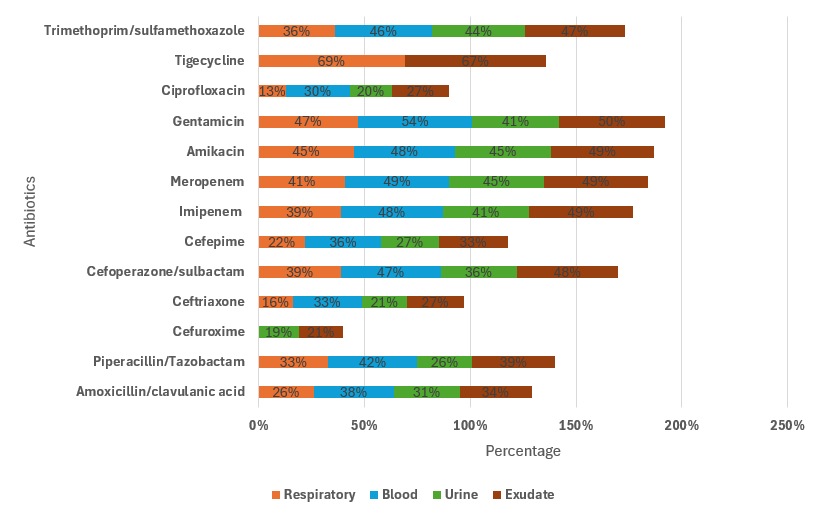


**Supplementary figure S1c: Year-wise antimicrobial susceptibility of *K. pneumoniae* isolated from various clinical sources in ICUs (2023)**


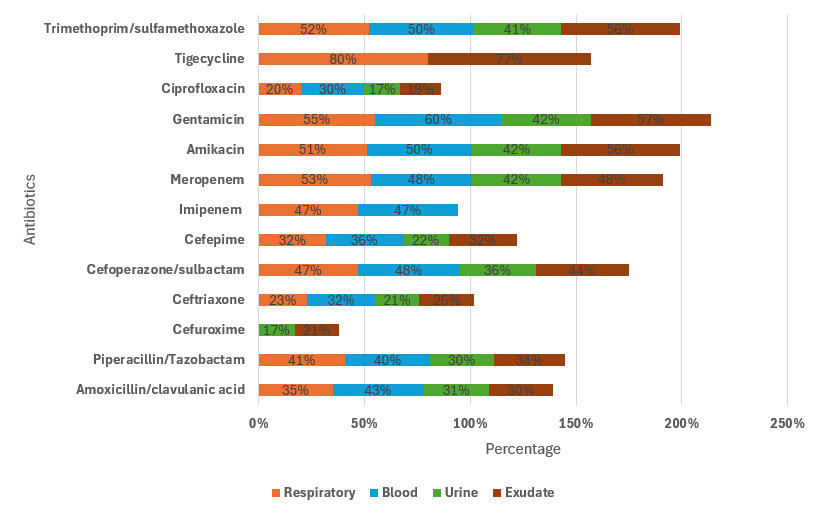


**Supplementary figure S1d: Year-wise antimicrobial susceptibility of *K. pneumoniae* isolated from various clinical sources in ICUs (2024)**


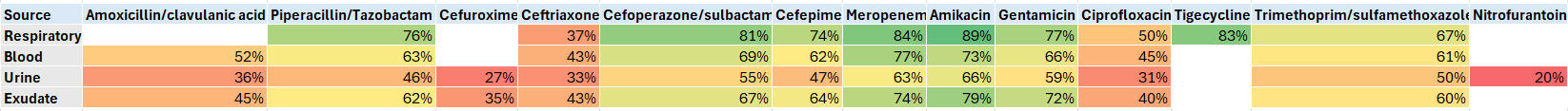


**2021:**


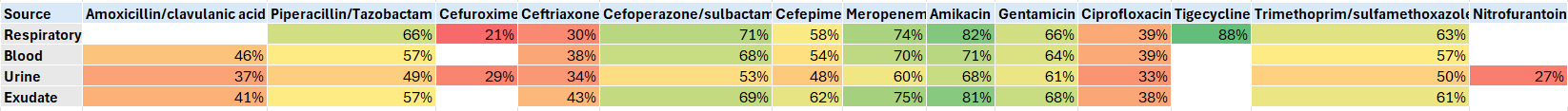


**2022:**


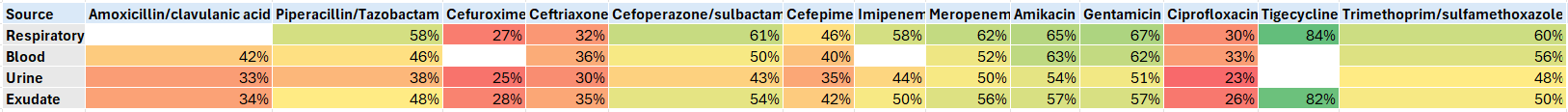


**2023:**


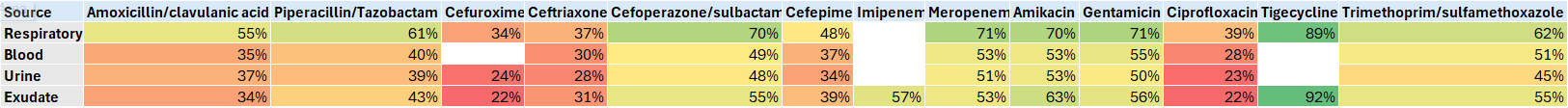


**2024:**

**Supplementary figure S2: Antimicrobial susceptibility heatmap of non-ICU *K. pneumoniae* isolates across clinical sources**

Each cell displays the percentage susceptibility of *Klebsiella pneumoniae* isolates to the corresponding antibiotic for the given year. The color gradient provides a visual representation of susceptibility levels, with red shades indicating lower susceptibility and green shades indicating higher susceptibility.

The percentage values represent antimicrobial susceptibility rates for individual antibiotics and are independent of each other; therefore, values do not sum to 100%.


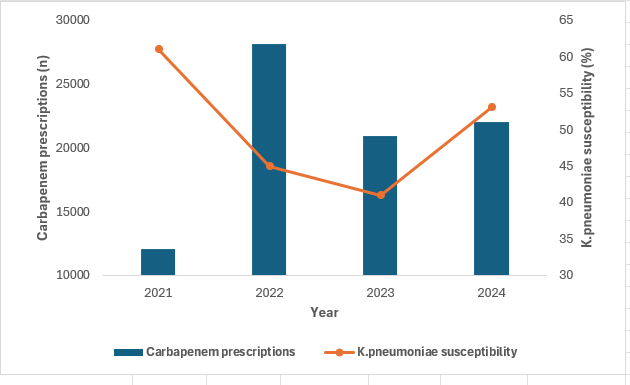


**Supplementary figure S3a: Temporal trends in Carbapenem utilization and *K. pneumoniae* susceptibility among ICU respiratory infections**


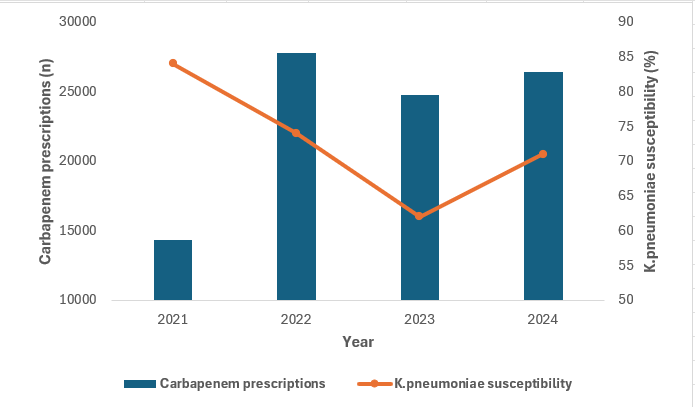


**Supplementary figure S3b: Temporal trends in Carbapenem utilization and *K. pneumoniae* susceptibility non-ICU respiratory infections**


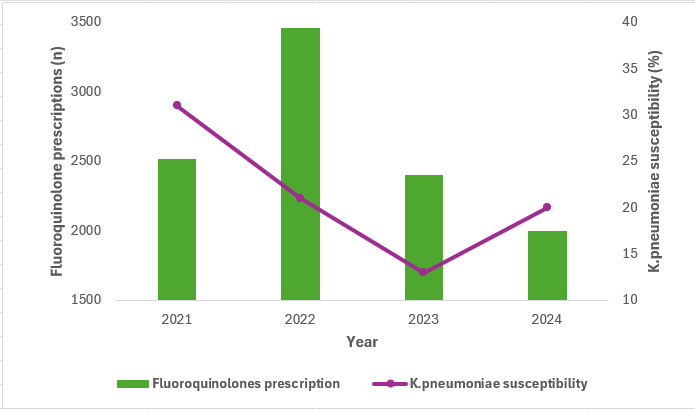


**Supplementary figure S4a: Temporal trends in Fluoroquinolones utilization and *K. pneumoniae* susceptibility among ICU respiratory infections**


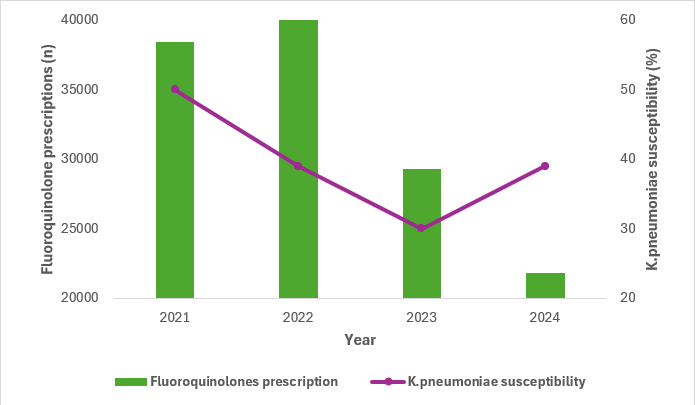


**Supplementary figure S4b: Temporal trends in Fluoroquinolones utilization and *K. pneumoniae* susceptibility among non-ICU respiratory infections**


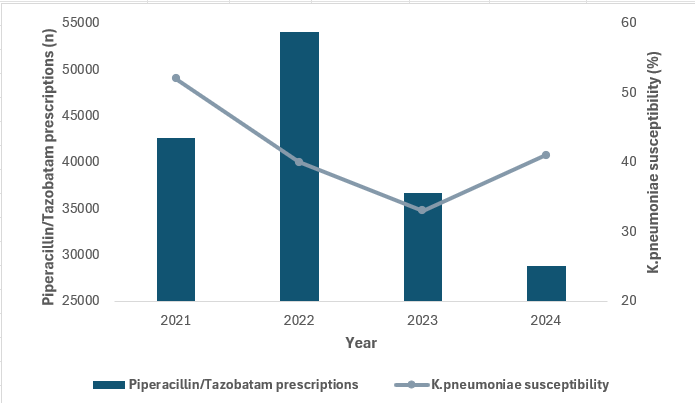


**Supplementary figure S5a: Temporal trends in piperacillin/tazobactam utilization and *K. pneumoniae* susceptibility among ICU respiratory infections**


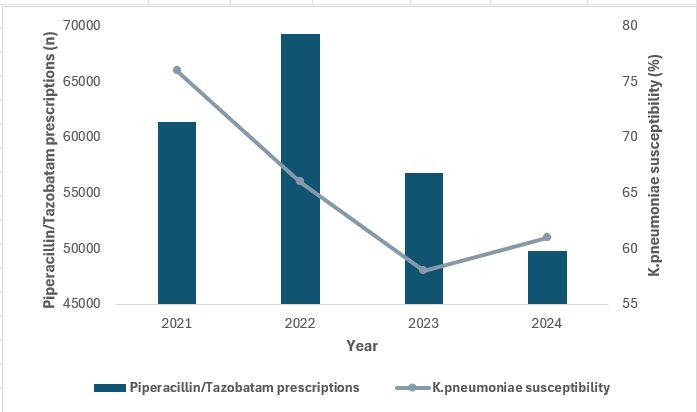


**Supplementary figure S5b: Temporal trends in piperacillin/tazobactam utilization and *K. pneumoniae* susceptibility among non-ICU respiratory infections**


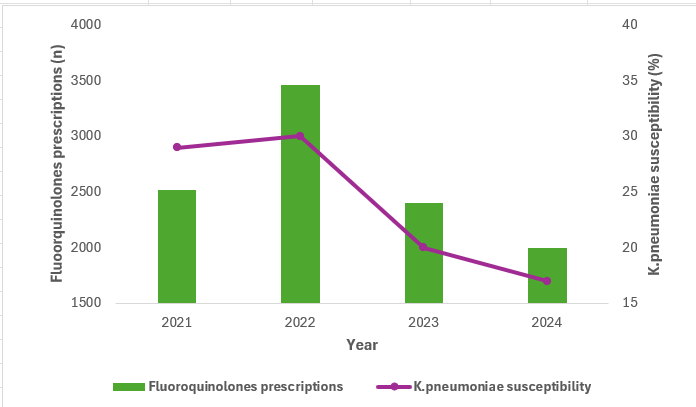


**Supplementary figure S6a: Temporal trends in fluoroquinolones utilization and *K. pneumoniae* susceptibility among ICU urinary tract infections**


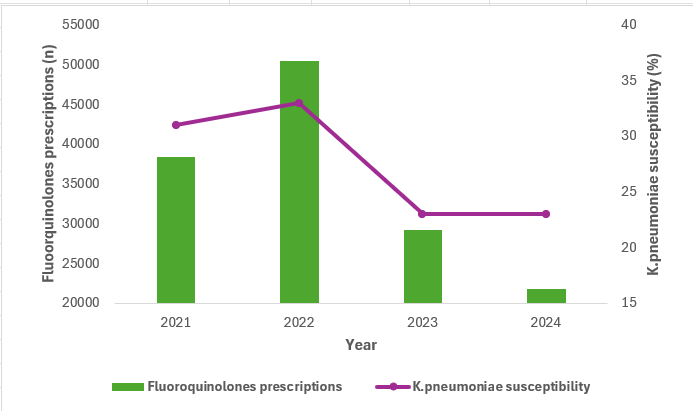


**Supplementary figure S6b: Temporal trends in fluoroquinolones utilization and *K. pneumoniae* susceptibility among non-ICU urinary tract infections**


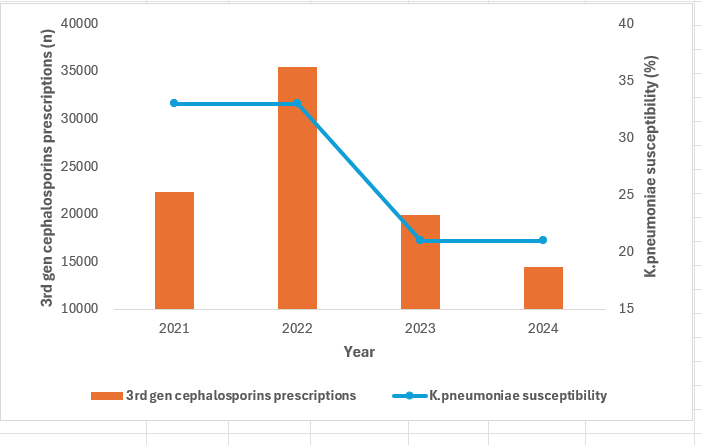


**Supplementary figure S7a: Temporal trends in 3^rd^ generation cephalosporins utilization and *K. pneumoniae* susceptibility among ICU urinary tract infections**


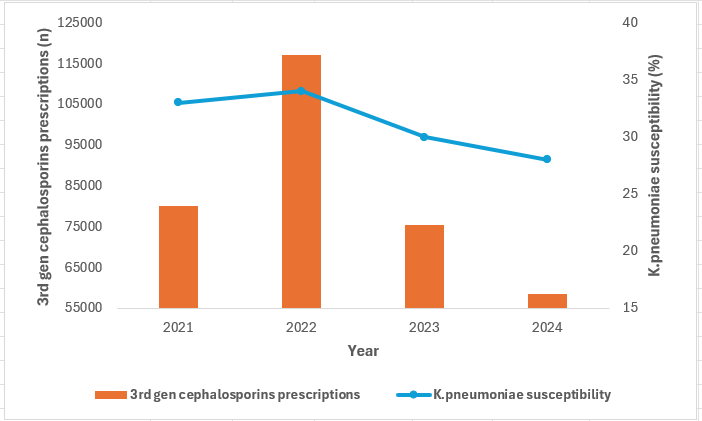


**Supplementary figure S7b: Temporal trends in 3^rd^ generation cephalosporins utilization and *K. pneumoniae* susceptibility among non-ICU urinary tract infections**


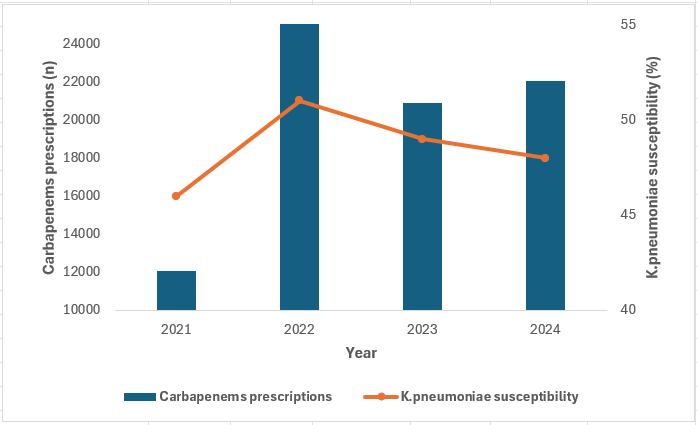


**Supplementary figure S8a: Temporal trends in carbapenems utilization and *K. pneumoniae* susceptibility among ICU exudates**


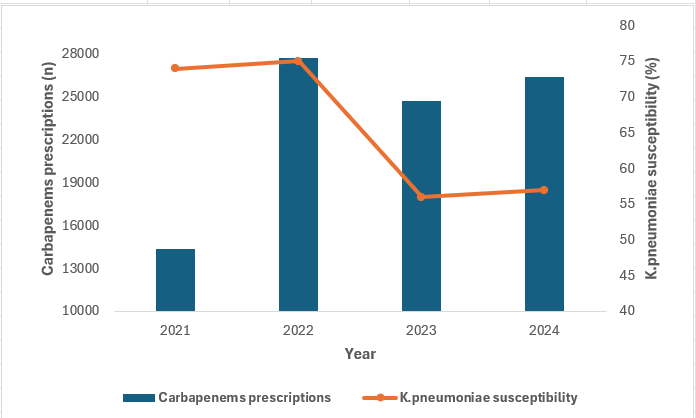


**Supplementary figure S8b: Temporal trends in carbapenems utilization and *K. pneumoniae* susceptibility among non-ICU exudates**

**
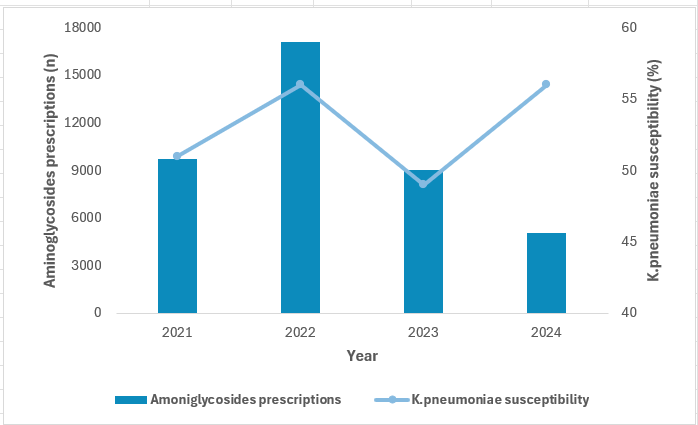
**

**Supplementary figure S9a: Temporal trends in aminoglycosides utilization and *K. pneumoniae* susceptibility among ICU exudates**

**
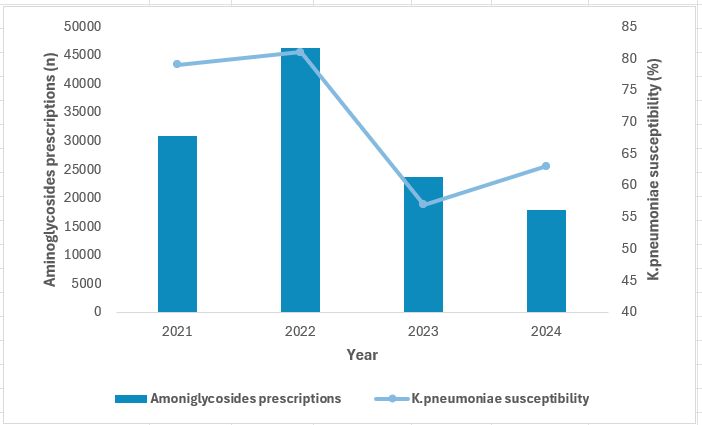
**

**Supplementary figure S9b: Temporal trends in aminoglycosides utilization and *K. pneumoniae* susceptibility among non-ICU exudates**


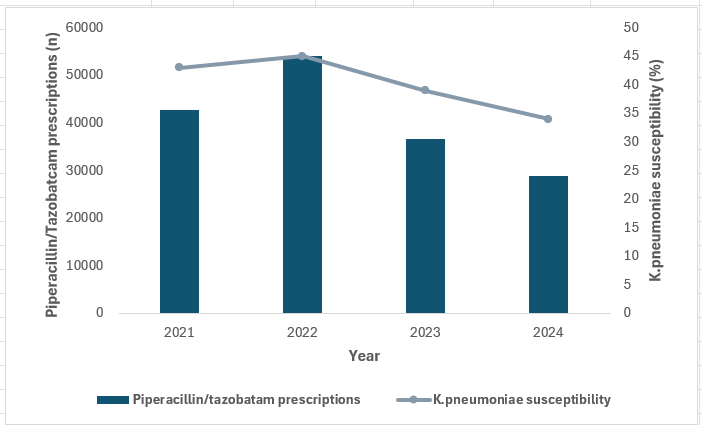


**Supplementary figure S10a: Temporal trends in piperacillin/tazobactam utilization and *K. pneumoniae* susceptibility among ICU exudates**


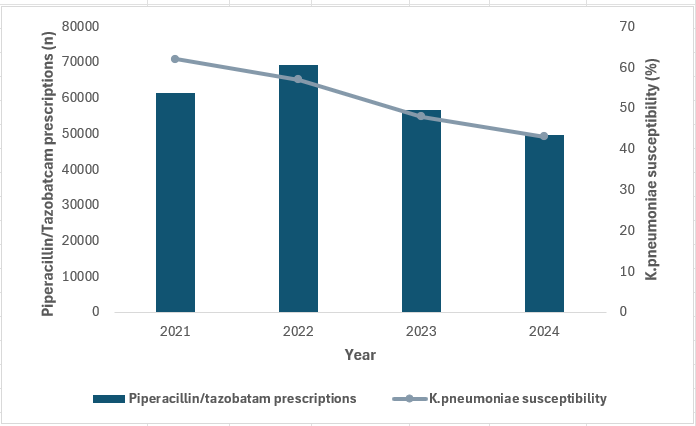


**Supplementary figure S10b: Temporal trends in piperacillin/tazobactam utilization and *K. pneumoniae* susceptibility among non-ICU exudates**

These dual-axis graphs illustrate temporal trends in antibiotic utilization and *Klebsiella pneumoniae* susceptibility over the study period. The graphical comparisons are descriptive and intended to visually explore temporal patterns; no formal statistical association between antibiotic consumption and susceptibility was tested.
